# Risk mapping of Rinderpest sero-prevalence in Central and Southern Somalia based on spatial and network risk factors

**DOI:** 10.1186/1746-6148-6-22

**Published:** 2010-04-28

**Authors:** Angel Ortiz-Pelaez, Dirk U Pfeiffer, Stefano Tempia, F Tom Otieno, Hussein H Aden, Riccardo Costagli

**Affiliations:** 1 Veterinary Epidemiology & Public Health Group, Department of Veterinary Clinical Sciences, The Royal Veterinary College, University of London, Hawkshead Lane, North Mymms, Hatfield, Herts, AL9 7TA, UK; 2 Terra Nuova East Africa, Raphta Road n.87 (Westlands), Maisonette 14/15, PO Box 74916, 00200 Nairobi, Kenya; 3 International Livestock Research Institute (ILRI), PO Box 30709, 00100 Nairobi, Kenya

## Abstract

**Background:**

In contrast to most pastoral systems, the Somali livestock production system is oriented towards domestic trade and export with seasonal movement patterns of herds/flocks in search of water and pasture and towards export points. Data from a rinderpest survey and other data sources have been integrated to explore the topology of a contact network of cattle herds based on a spatial proximity criterion and other attributes related to cattle herd dynamics. The objective of the study is to integrate spatial mobility and other attributes with GIS and network approaches in order to develop a predictive spatial model of presence of rinderpest.

**Results:**

A spatial logistic regression model was fitted using data for 562 point locations. It includes three statistically significant continuous-scale variables that increase the risk of rinderpest: home range radius, herd density and clustering coefficient of the node of the network whose link was established if the sum of the home ranges of every pair of nodes was equal or greater than the shortest distance between the points. The sensitivity of the model is 85.1% and the specificity 84.6%, correctly classifying 84.7% of the observations. The spatial autocorrelation not accounted for by the model is negligible and visual assessment of a semivariogram of the residuals indicated that there was no undue amount of spatial autocorrelation. The predictive model was applied to a set of 6176 point locations covering the study area. Areas at high risk of having serological evidence of rinderpest are located mainly in the coastal districts of Lower and Middle Juba, the coastal area of Lower Shabele and in the regions of Middle Shabele and Bay. There are also isolated spots of high risk along the border with Kenya and the southern area of the border with Ethiopia.

**Conclusions:**

The identification of point locations and areas with high risk of presence of rinderpest and their spatial visualization as a risk map will be useful for informing the prioritization of disease surveillance and control activities for rinderpest in Somalia. The methodology applied here, involving spatial and network parameters, could also be applied to other diseases and/or species as part of a standardized approach for the design of risk-based surveillance activities in nomadic pastoral settings.

## Background

Somalia's livestock production sector accounts for at least 40% of the gross domestic product (GDP) with 55% of the human population being directly involved in the rearing of livestock [1]. Pastoral movements across Somalia's borders for the purposes of grazing livestock and livestock-related trade have occurred for centuries [2], resulting in seasonal movement patterns of herds/flocks in search of water and pasture. Moreover, in contrast to most pastoral systems, which are normally aimed at household subsistence, the Somali livestock production system is oriented towards domestic trade and export [3]. Livestock are shipped to various countries in the Arabian Peninsula, and trekked or transported to markets in Kenya, Djibouti, and Ethiopia [4]. In 2007 some 1,639,625 heads of cattle, sheep, goats and camels were exported through Bossasso port and a total of 1,633,793 through Berbera port [4], the two major export markets in the country.

Despite the collapse of the Somali government in 1991 and the lack of continued delivery of public services, an informal system of stateless order, social trust and an informal economy have allowed rural and urban populations to survive unfavourable economic and political circumstances [5]. But the Somali livestock industry is therefore now even more vulnerable to the introduction of bans by importing countries for two reasons. One being the stringent measures for livestock trade specified under the Sanitary and Phytosanitary (SPS) Agreement of the World Trade Organization http://www.wto.org, of which Somalia is not a member, and the other the poor standards of veterinary services and the absence of control measures for fighting trans-boundary animal diseases. The high mobility of the livestock population poses an additional challenge for the control and establishment of credible certification systems for the major trans-boundary diseases occurring in Somalia. An understanding of the aggregation/dispersion mechanisms and contact structure of the Somali livestock potentially could assist in setting up appropriate spatial risk-based surveillance activities and control measures that may lead to the establishment of an internationally accepted certification system for this nomadic pastoral livestock production system.

Data on the geographical patterns of human and animal population distributions are now commonly considered in epidemiological studies. High density of susceptible populations has been shown to be a key factor in the transmission of infectious diseases in humans [6]. In the case of animal populations, spatial proximity is closely linked to the transmission of many infectious diseases [7]. The geo-referencing of animals kept in herds through the point location of the farm is relatively simple and cost-effective [8]. However in settings where mobility is a major feature of the husbandry systems the geographical location of animals is no longer a discrete entity hence alternative approaches are required for the collection and analysis of their spatial data.

Network data are not usually linked to geographical data, and social network analysis rarely considers the spatial configuration of the links [9] apart from the pure visualization of the networks. Definitions of links within networks based on spatial criteria such as the distance between pairs of nodes, usually estimated using the Euclidean distance ("as the crow flies") are rarely available in the literature. For example, Webb [10] made the assumption in her analysis that there was a link between two farms if their postcodes were less than 25 km apart, as a proxy for the catchment area of farm animal veterinary practices. Dent et al. [11] considered poultry premises to be linked if they were within 3 km distance from each other.

The purpose of epidemiological modelling of spatial data is to explain or predict the occurrence of disease [12-18]. The production of risk maps can guide decision makers provided that the underlying assumptions of uncertainty and variability are exposed [19]. Using the data from a previous rinderpest survey conducted in the Central and Southern regions of Somalia, it was possible to explore the topology of a contact network of cattle herds based on a spatial proximity criterion and other attributes related to cattle herd dynamics. The objective of the current study is to integrate spatial mobility and other attributes with GIS and network approaches in order to investigate their effect on disease presence and develop a predictive spatial model of presence of rinderpest. The risk map generated by the predictive model can be used to inform the design of risk-based surveillance activities of rinderpest by identifying high-risk point locations where to prioritize disease surveillance and control activities. Surveillance efforts could be targeted at high-risk areas which would lead to more effective use of the scarce veterinary resources in Somalia, and ultimately, contribute to the establishment of an internationally credible certification system for livestock export by nomadic pastoral systems. The standardisation of the proposed methodology for cattle will serve as a model for other livestock species (e.g. small ruminants and camelids) and other diseases.

## Methods

### Rinderpest data

**Figure 1:**
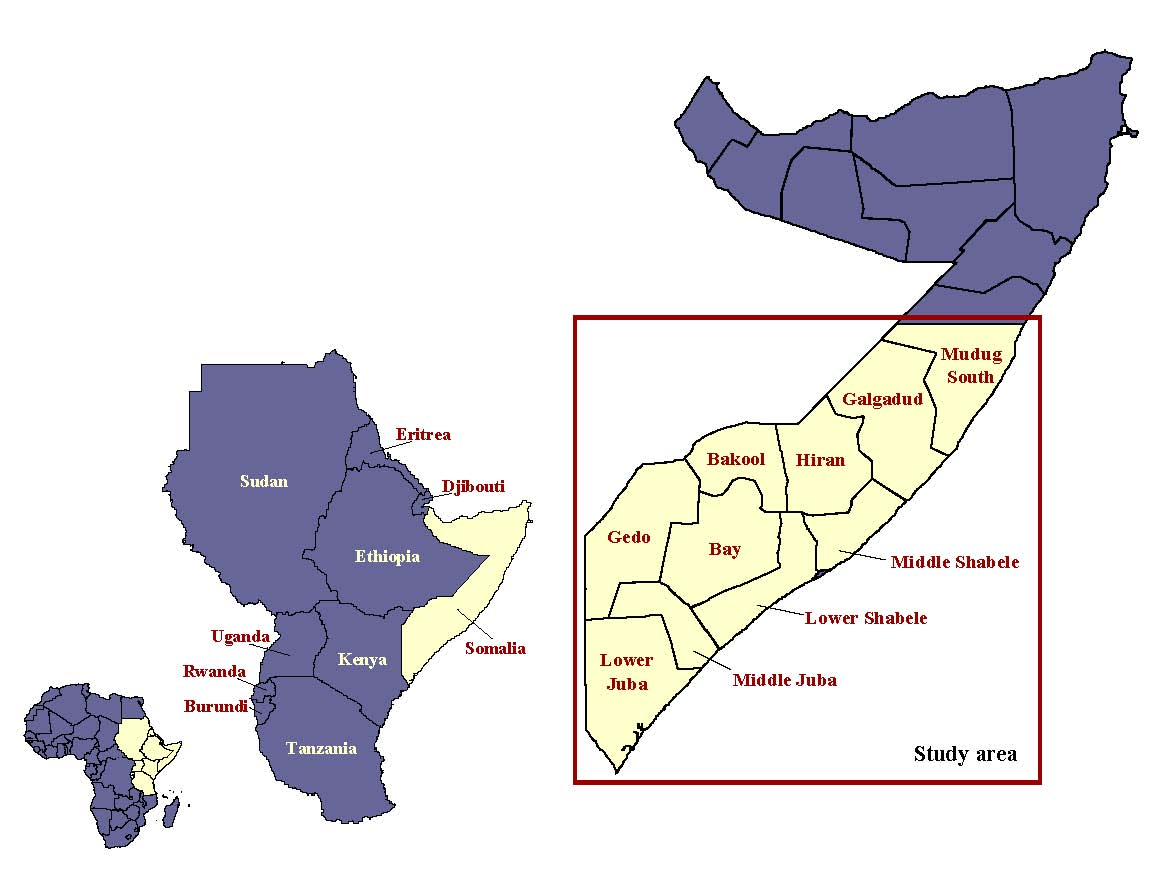
**Map of Somalia with the names of the Central and Southern regions included in the study area**.

A cross-sectional survey was carried out in Central and Southern Somalia to estimate the prevalence of rinderpest (RP) in 2002-2003. The study area covered ten administrative regions of Central and Southern Somalia: Mudug, Galgadud, Hiran, Middle Shabele, Lower Shabele, Bay, Bakool, Lower Juba, Middle Juba and Gedo (Figure 1). Ninety percent of Somalia's cattle population are kept in these regions [20]. Details of the design and implementation of the survey are described elsewhere [21].

At selected sampling sites, a minimum number of 15 eligible animals were sampled per herd and questionnaires were administered to the livestock owners of the sampled herds in order to collect data on species, herd/flock size and location during each climatic season over the two years prior to the date the survey was conducted in that point and livestock markets (primary and secondary) where the pastoralists sold their animals during the same period. A total of 9216 serum samples were collected from cattle aged 1 to 3 years at 562 sampling sites. In addition, 1071 sera were collected from cattle older than 3 years at 58 sampling sites. All serum samples collected were tested for the presence of RP antibodies using a RP Competitive Enzyme Linked Immunosorbent Assay (C-ELISA) directed against the H protein of the virus [22]. All samples having a percentage of inhibition (PI) above 50% were considered positive in accordance with the OIE recommendation for testing large numbers of sera.

### Home range

**Figure 2:**
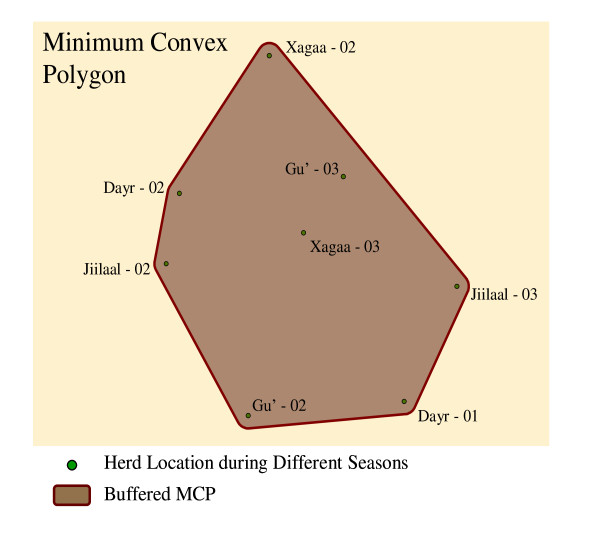
**Example for the calculation of the home-range using the Minimum Convex Polygon (MCP) method for one of the point locations of the Rinderpest survey**.

The home-range (Hr) of the 562 point locations where at least one cattle herd was located was estimated by first obtaining the geographical coordinates [23,24] for each of the points where the herd/s had been reported during the surveyed seasons (Jiilaal: Dec. - Mar.; Gu': Apr. - Jun.; Xagaa: Jul. - Sep. Dayr: Oct. - Nov.) over the two years prior to the time of the survey. If a particular location was not included in the available databases then the location was visited and the geographical coordinates were recorded. The home range for each point was then calculated based on these point locations using the minimum convex polygon (MCP) method [25] and adding a buffer of 5 km around the MCP in order to account for the daily mobility of the herds. The area (in km2) of the MCP represents the estimated home range for a particular point based on the observed movements of the herd/s in that point. Figure 2 displays the location of a subset of locations for a herd and the resulting MCP polygon from which a circular home range was calculated.

**Figure 3:**
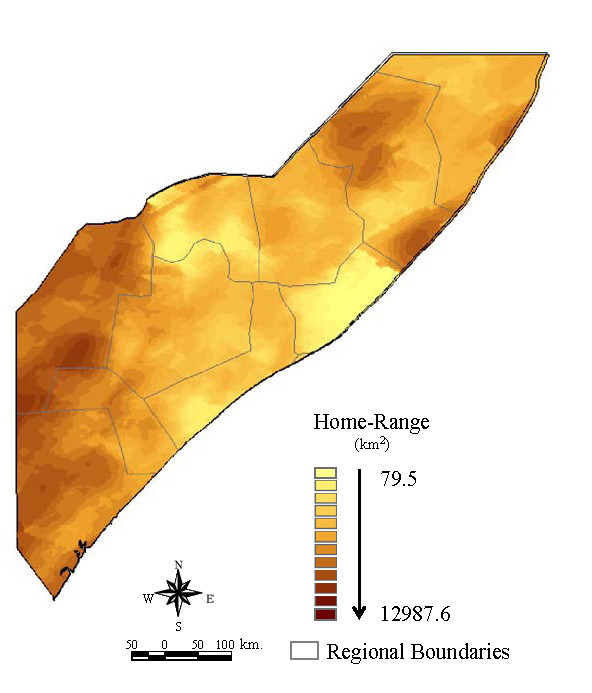
**Cattle home range distribution map of the study area obtained via the kriging method**.

A map representing the spatial variation in home range area (km^2^) for cattle herds was then generated using kriging techniques [26-29]. Kriging is a group of geostatistical techniques for interpolating the value of a random field at an unobserved location based on observed values at nearby locations [28]. The home range values (z) for each point included in the study were used to generate the kriged surface utilizing as location the coordinates (x, y) of the centroid of the home-range polygon estimated for each individual herd. The kriged surface representing the average home range estimates at each location was created by first fitting an n^th ^order polynomial surface to the data [30]. Akaike's Information Criteria (AIC) was used to compare the fit of the different surfaces from 1^st ^to 4^th ^polynomial order and different covariance functions (Gaussian, exponential, spherical and Matern) [31]. Then, the covariance structure of the data was modelled using a exponential covariance function with a range value of θ = 0.24 and a nugget of α = 0.35. The final surface was generated by kriging the residuals of the surface using the selected exponential covariance function. The kriged map of the spatial pattern of average cattle home range is displayed in Figure 3.

Attributes for the 562 point locations were generated through spatial queries and analysis in ArcGIS 9.2. (^© ^ESRI), using as unique identifier a point location reference number linked to the projected coordinates. Seven attributes were extracted for each point location. (1) The type of point (categorical): villages, towns [24], water points [23], primary/secondary markets [32], and grazing points [33]. (2) Proximity to roads (binary): less than 500 meters from a main road. (3) Proximity to trade routes (binary): the locations of three major trade routes were recorded in southern and central Somalia by Terra Nuova [32] by use of a radio collar GPS fitted to an animal within the traded herds being trekked from secondary markets to export points. The proximity was based on whether a point's estimated home range as described above intersected the trade routes. (4) Speed at trade route (numeric): the speed of the movement at each trade route point was calculated for segments of time and distance between points along the trade routes derived from the spatial join of collar (cattle) and drover (handler) GPS data and then aggregated based on drover GPS locations. If the point was linked to a trade route, speed in km/h was included. (5) Home range radius (numeric): radius of the home range assuming circular shape (method described above). (6) Herd density (numeric): herd density values assigned to the survey points through intersection of the points with a distribution map of herd density generated through moving average neighbourhood analysis [31] from livestock census data [20]. (7) Herd size (numeric): herd size values assigned to the survey points through intersection of the points with a distribution map of herd sizes [21].

### Network

**Figure 4:**
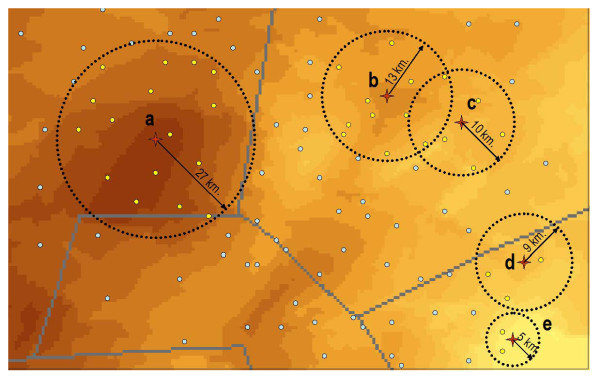
**Graphical representation of the process used for generating the herd movement contact network**. Points b and c and d and e are linked. Point a is not linked to b, c, d or e.

The contact network between nomadic herds was constructed using the 6738 point locations of towns, villages, watering points and grazing areas, including the 562 points from the survey, extracted from the above-mentioned databases. The home range for each of the 6738 points was assigned by spatially overlaying the point location map with the interpolated home range map shown in Figure 3. Once all points had been assigned a home range, home radii were calculated assuming a circular shape for the home ranges. Thereafter a Euclidean distance matrix between each pair of points was generated using the Hawth's Analysis Tool for ArcGIS http://www.spatialecology.com/htools/. If the distance between a pair of points was less than the sum of their home range radii then these two points were linked by a symmetric unvalued link. Thus the links between nodes in the network were derived from contact (Points d and e in Figure 4) or overlap of the points' home ranges (Points b and c in Figure 4). A graphic representation of the comparison of home range radii and distances between points to generate the network is illustrated in Figure 4. The following general parameters of the symmetric binary network were extracted: number of nodes, number of links, density, giant weak component and average distance among reachable pairs. For individual nodes, the following centrality measures were extracted: degree, betweenness, closeness and clustering coefficient.

### Spatial logistic model

The study population comprised the initial 562 randomly selected locations included in the cross-sectional rinderpest survey, for which data on number of animals sampled and test results were available. If we consider the number of positive samples for Rinderpest at point *i *out of *N*_*i *_sampled animals, *Y*_*i *_is a binomial random variable *Y*_*i *_~Bin (*N*_*i*_, *p*_*i*_) with *p*_*i *_being the prevalence of infection at each point. The spatial binomial logistic model is given by:

where *β*_0 _is the intercept, *x*_*i *_the covariates, *β*_*i *_the regression coefficients, *ξ*_*i *_normally distributed non-correlated residuals and *S*_*i *_the spatial component (spatial autoregressive coefficient), which is assumed to have zero mean, variance *σ*^2 ^and an isotropic covariance function *ρ *(*d*_*ij *_*θ*), where *θ *is the range coefficient and measures the decay of the spatial autocorrelation and *d*_*ij *_is the Euclidean distance between each pair of points.

A generalized linear mixed model with a logit link function was fitted. The outcome variable was the count of rinderpest positive samples expressed as a binomial distribution to account for the variation in the number of animals tested. The following covariates were introduced in the multivariable model as fixed effects: type of point, next to road, linked to trade route, speed at trade route, herd density, herd size, home range radius, degree, betweenness, closeness, inclusion in the giant weak component and clustering coefficient. An exponential spatial covariance structure (exp (-*d*_*ij *_/*θ)) *was included as a random effect. The analysis was implemented using restricted pseudo likelihood with the GLIMMIX procedure of SAS 9.1 (^© ^2002-2003 by SAS Institute Inc., Cary, NC, USA). Linearity of the effects was tested by categorising continuous variables into three-category variables. The statistical significance of the fixed effect variables was determined using Type III tests. The ability of the binomial model to predict the outcome was estimated using as cut-off point the mean of the fitted probabilities predicted by the model in order to transform them into a binary outcome [34]. Predicted probabilities below the cut-off value were considered as predicting absence (0) and probabilities above the cut-off value were considered predicting presence (1).

Once the final model was fitted, a semivariogram of the residuals was generated using the ArcGIS 9.2 Geostatistical Analyst extension (^© ^ESRI) for assessing the potential presence of spatial autocorrelation in the model residuals. The logistic regression model equation was applied to the set of 6176 point locations using their specific covariate patterns. Attributes for the significant variables of the final model were extracted for the set of point locations using the same methods as for the survey points. The predicted prevalence using the final model equation for each point location was exported to ArcGIS 9.2 (^© ^ESRI) for visualization of the resulting risk map. A kernel smoothed intensity map was produced with 50 km bandwidth and 5 km grid cells using the Spatial Analyst extension of ArcGIS 9.2 (^© ^ESRI).

## Results

**Figure 5:**
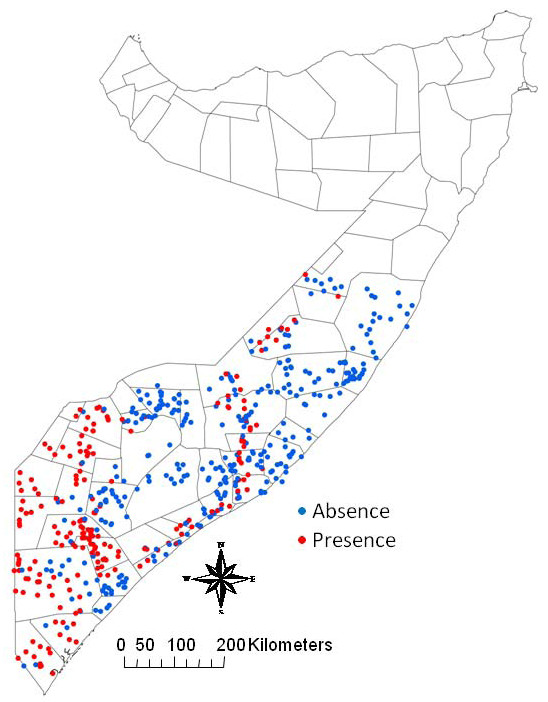
**Distribution of the 562 point locations of the rinderpest survey and their serological status (Presence/Absence)**.

The 562 point locations included in the survey consisted of 479 villages (85.3%), 6 towns (1%) and 77 grazing points (13.7%). Fifty-five of the points were linked to at least one of the three trade routes (9.8%). The average size of the home range radii was 23 km (STD: 13.8 Range: 0-97 km). The average herd density across the points was 13.3 herds (STD: 6.5 Range: 4-24) and the average herd size was 45.6 (STD: 27.5 Range: 10.4-164.5). The number of point locations with rinderpest confirmed in at least one of the animals sampled was 206 (36.6%) with a mean prevalence among positive points of 18.2% (Range: 0.18-82.4%). The distribution of the point locations of the rinderpest survey and their disease status are displayed in Figure 5.

The final model was fitted using data for 562 point locations and contains three continuous-scale variables significant at P < 0.05 level: home range radius, herd density and clustering coefficient. The regression coefficients of the model, their standard errors, P values and the corresponding odds ratios with 95% confidence intervals are shown in Table 1. An increase of 1 km in the home range radius increases the risk of having rinderpest by 1.6%. For an increase of one herd in the herd density at a point location the risk of having rinderpest increases by 12%. The risk of serological evidence of rinderpest being present amongst the animal sampled at a point location with maximum clustering coefficient (1) is nearly 20 times the risk of a point whose linked points are not connected with each other. All Type III effects for the variables included in the final model were significant at P < 0.05 level. Predicted prevalence for each point location was converted into a dichotomous presence/absence outcome using the mean of the fitted continuous-scale probability values as the cut-off; predicted probabilities less than 0.126 were classified as absence of rinderpest and those greater than 0.126 were classified as presence of rinderpest. The sensitivity of the model is 85.1% and the specificity 84.6%, correctly classifying 84.7% of the observations. Table 2 shows the comparison between fitted and observed values.

**Table 1:** Coefficients of the final binomial generalized mixed model (GLM) with standard errors, odds ratios and 95% confidence intervals

	Coefficient	Standard Error	P-value	Odds Ratio (95% CI)
Intercept	-7.28	0.68	P < 0.001	
Home range radius (in Km)	0.016	0.004	P < 0.001	1.016 (1.008-1.025)
Herd density	0.11	0.02	P < 0.001	1.12 (1.06-1.18)
Clustering coefficient (cc1)	2.97	0.72	P < 0.001	19.6 (4.7-81.8)

**Table 2:** Predicted outcomes from the final binomial generalized mixed model (GLM) compared to the observed data

		Observed		
		Presence	Absence	Total
Predicted*	**Presence**	80	72	152
	**Absence**	14	396	410
	**Total**	94	468	562

*Presence: if the observed prevalence or the predicted probability are greater than the mean of the distribution of the fitted probabilities of the model. Absence if they are lower

**Figure 6:**
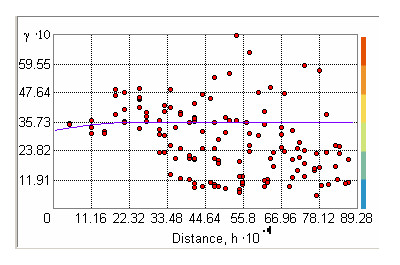
**Semivariogram of the residuals of the final binomial GLM model. X axis: distance of the space intervals (h)**. Y axis: semivariance (γ)

The variance of the underlying residual spatial effect is 0.003, and the distance at which the correlation between point locations has decreased to the 0.05 level is 1.5 km. Visual inspection of the semivariogram of the model residuals as displayed in Figure 6 suggests the absence of spatial autocorrelation.

The network contains 6738 nodes and 319,887 links with a density of 1.4% and an average degree of 224.68 (range: 1-605). The average distance between reachable pairs is 7.7 links with the maximum distance between two points being 24. There is a giant weak component containing 2950 nodes (43.78%). Within the network the 562 points of the survey had an average degree of 169 (STD: 118 Range: 2-605), average betweenness of 0.001 (STD: 0.004 Range: 0-0.05), average closeness of 0.12 (STD: 0.023 Range: 0.08-0.17) and average clustering coefficient 0.67 (STD: 0.01 Range: 0.3-1).

**Figure 7:**
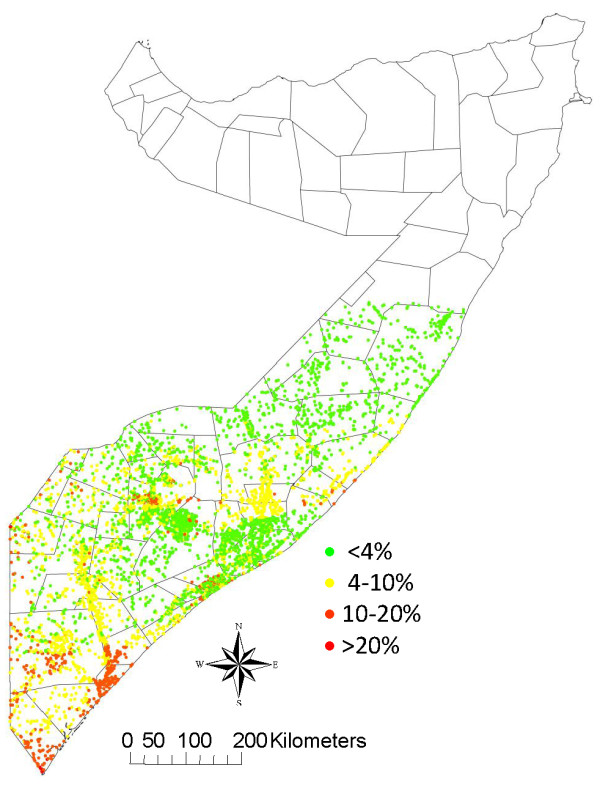
**Risk map of the 6176 point locations in the study area based on the probability of Rinderpest presence according to the predictive model**.

**Figure 8:**
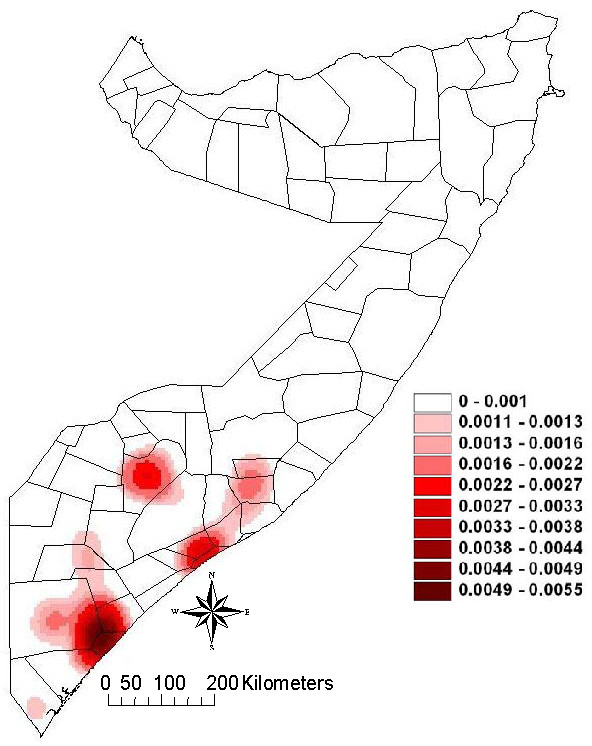
**Kernel smoothed intensity map of the predicted probability of Rinderpest presence according to the predictive model (5 km grid cells, 50 km bandwidth)**.

The risk map for the 6176 point locations is shown in Figure 7. Predicted prevalence of rinderpest has been aggregated into four categories for visual display in different colours: <4%, 4-10%, >10-20% and >20%. The kernel smoothed intensity map in Figure 8 shows predicted prevalence using 5 km square grid cells. Assuming the equivalence of predicted prevalence and probability of presence of rinderpest, the areas at high risk of having serological evidence of rinderpest amongst the animals sampled, as predicted by the model, are located mainly in the south of the study area, in the coastal districts of Lower and Middle Juba, the coastal area of Lower Shabele and in the regions of Middle Shabele and Bay. There are also isolated spots of high risk along the border with Kenya and the southern area of the border with Ethiopia. Very low risk areas are situated in the central and northern regions of the study areas, in the regions of Galgadud and Mudug, with low risk areas in the regions of Bay, Gedo and both inland Middle and Lower Shabele.

Evaluating the model by comparing the predicted high-risk areas as shown in the point location map (Figure 7) and the kernel smoothed intensity risk map (Figure 8) with the findings of the rinderpest survey (Figure 5), the model appears to correctly predict the presence of rinderpest in Lower and Middle Juba and Middle Shabele. The model predicts Gedo and Western Galgadu on the border of Ethiopia as low risk, both of which are provinces where rinderpest was confirmed during the survey. On the other hand, the model predicts high risk at point locations in areas where the survey did not find positive cases, such as the border area between Bakool and Bay. It is worth stressing the difference in the scale of risk between the risk point location and the kernel smoothed intensity map where the colour grade in the latter determines the risk for 25 km^2 ^raster cell areas at much lower scale than the point map.

## Discussion

This study has applied spatial and network analysis techniques to a combination of observed and estimated data with the objective to produce a predictive model for the presence of serological evidence of rinderpest in the southern and central regions of Somalia. Incorporating the network framework into GIS has been attempted before mainly in geography and social sciences. For example, Faust et al. [9] showed how social relations can be determined by geographical location and other spatially-related attributes. In the context of disease transmission, Rothenberg et al. [6] demonstrated that social and geographical distance can support transmission of disease. Following the same rationale the main hypothesis tested in this study was whether the presence of rinderpest is associated with the network centrality related to its geographical location and/or to other attributes of the geographical locations. The results have shown the significance of both types of factors. One of these is the degree of interactions between herds due to overlapping home ranges in neighbouring areas, expressed by the clustering coefficient of the nodes derived from the spatial contact network. The other is the home range radius or area where nomadic herds graze throughout the year and the herd density are both significant risk factors. The combination of the three significant factors reveals the importance for the presence of rinderpest of local neighbourhood and geographic compactness interacting in densely populated areas, an effect that has been reported before for human diseases such as STDs and HIV-AIDS [35,6].

Network parameters that take into account paths between all possible pairs of nodes such as betweenness and closeness did not appear to be significantly associated with the presence of disease. Large intermediaries or globally relevant nodes in the network represented by high betweenness and closeness are not roles that increase the risk of disease presence in the population of the survey, nor is the membership in within-network structures such as the giant weak component. Although two nodes/points far apart can be linked via a path of overlapping home ranges, being reachable by many other nodes in the network, in between many other shorter paths, or included in a connected subpopulation of nodes, the results suggest that the amount of overlap between neighbouring herd home ranges is the only network feature that substantially increases the risk of rinderpest. The spread of rinderpest to distant areas may follow other paths unaccounted for in this study. For example, the effect of markets has not been addressed in this analysis. It would be advised to investigate additional potential risk factors such as presence of or distance to local or regional markets. Nor has the possible risk attributed to long-distance movements via trade routes been assessed given the presence in the dataset of only three trade routes linked to each other and located in the southern districts of the study area. Incorporating more comprehensive data on putative risk factors such as the movement of the herds to markets and borders with neighbouring countries would allow testing these hypotheses.

The inclusion of variables with a strong spatial component in the multivariable analysis required the application of an analytical method that could account for the dependence of the network data due to both the inherent nature of the network data and the spatial definition of the link. Thus, the general assumption of dependence within the network data analyzed is confirmed by both the network parameterization and the spatial nature of the link. In fact, the spatial component of the binomial generalized linear mixed model, the spatial autoregressive coefficient, is similar to the local and interaction effects in network models [36] in what Anselin [37] called the family of autocorrelation models. In both cases the specifications of the chosen weight matrix are important for testing the statistical significance of a particular parameter. In this case, the spatial correlation function accounts at the same time for the similarity of home range radius and herd density between proximal points and the similarity of clustering coefficient of points linked in the network.

The internal validation of the final model showed a very high predictive ability. Its application to a different dataset has produced risk maps of point locations and areas based on the model estimates. In general, the location of the high-risk points agrees with the observed points positive to rinderpest in the original dataset, which suggests that the areas affected by rinderpest are characterised by large home ranges, large herd density and strong local interaction between neighbouring nomadic herds. However there is an area in Gedo region close to the border with Ethiopia and Somalia where the risk map predicts points of high prevalence whereas the kernel smoothed intensity map does not. This is due to the presence in that area of a small number of points with very large home ranges and high herd density that increase the risk at point level but dilute the risk at area level. This is a methodological constraint rather than an incoherent result given the difficulty of obtaining stable risk estimates if intensities vary substantially across an area [7].

Both, the point and area risk maps, identified a high risk area that the survey sampled at low intensity and did not detect rinderpest. It is located in the northern part of the Bay region close to the border with Bakool. These areas of predicted high risk and for which disease data is sparse or absent and were not well covered by the survey should therefore be prioritised for surveillance purposes.

The risk maps have identified areas in the Central and Southern regions of Somalia where veterinary authorities should concentrate their efforts for implementation of surveillance activities. It is recommended to validate these results with local experts and through further surveys to ascertain the rinderpest status in areas of predicted high risk where the disease is not currently known to be present. In this respect similar serological studies subsequently conducted in the study area detected rinderpest antibodies in the high-risk areas identified under this study. However, the observed sero-prevalence in these areas was observed to decline over time [38-40]. Similar sero-prevalence studies conducted in neighbouring areas of Kenya and Ethiopia in subsequent years also showed a very low sero-prevalence or the absence of rinderpest antibodies [41]. The progressive decline in rinderpest antibody prevalence is consistent with virus extinction. The verification of freedom from infection from the Somali Eco-System is currently in progress.

This methodology can also be used for other diseases and/or species as part of a standardized approach for the design of risk-based surveillance activities in nomadic pastoral settings. The detection of high risk areas and hot spots will assist veterinary authorities in strengthening the veterinary surveillance in such areas in order to apply control measures at the local level and to minimize the potential spread of the disease to other regions. The application of disease control measures based on geographical factors and applied at local/compartmental level could be a starting point for the establishment of an internationally recognised certification system in settings where large-scale surveillance and control activities cannot be implemented.

## Conclusions

This study demonstrates the potential for the integration of different data sources and analytical methods for the development of a spatial and network predictive model of the presence of rinderpest in central and southern Somalia. The identification of point locations and areas with high risk of presence of rinderpest and their spatial visualization as a risk map can assist in prioritizing disease surveillance and control activities for rinderpest in Somalia.

Although considerable progress on RP eradication has been achieved in the study area since this study was conducted, the value and application of the applied methodology remains of interest for the investigation of livestock diseases in nomadic and semi-nomadic pastoral systems. This methodology can be used as part of a standardized approach for developing new decision-support tools for the design of risk-based surveillance activities in nomadic pastoral settings where the particular husbandry system, the lack of infrastructure and the inadequacy of the available veterinary services prevent the implementation of comprehensive control policies.

## Competing interests

The authors declare that they have no competing interests.

## Authors' contributions

AOP designed the study, conducted the network and multivariable statistical analysis and wrote the manuscript. DUP conducted the multivariable statistical analysis and reviewed the manuscript. ST conducted the rinderpest survey, designed the study and conducted the analysis of estimation of home range. FTO collected and managed data and reviewed the manuscript. HHA conducted the rinderpest survey and RC reviewed the manuscript. All authors read and approved the final manuscript.
